# Medicines as Disturbing Agents

**Published:** 1869-11

**Authors:** 


### Editorial Department.

### Medicines as Disturbing Agents.

It is very natural for physicians to be constantly alert to discover or learn the curative properties of drugs, but the attention is not as strongly directed to the possible disturbing influences which they possess. We are generally aware that even the most valuable remedies do, in certain cases, produce some ill effects; but we are scarcely alive to the fact, that almost all medicinal agents do harm unless they are so strongly indicated, for the removal of disease, that the good greatly overbalances the injury they produce. How is this? let us examine into the case a little in detail. Take, for instance, our most valuable medicine. What are the ill effects of opium? It disturbs digestion, constipates the bowels, diminishes the desire and relish for food, lessens many of the natural secretions, and in various ways acts as a disturbing element when introduced in any form into the system. Opium then, our most valuable medicine, is in some degree productive of harm—often of much more good than harm, but almost always of some harm—even in the most favorable cases, to say nothing of the instances where this drug produces peculiarly unpleasant effects. Opium, in its various forms, is probably worth more in the treatment of disease, or the proper care of the sick, than any other drug; it stands at the head in its range of application and in its curative influence, but it is not without its ill effects, which are temporary and unimportant, and also effects more permanent and positively injurious. All the other narcotics are liable to produce equally disturbing effects in proportion to their capacity for good. If we pass on to mercury, the remedy next in rank and influence, all will at once appreciate its power for evil—some may even go so far as to deny its capacity for good—but, we think, even balanced minds will not take ground wholly against it, while all will admit that it is a disturbing element in the economy, not naturally a component part of the animal tissues, and soon eliminated if introduced.

Iodine is another of our valuable remedies, but its influence for either good or evil is not so manifest. It enjoys a reputation for stimulating the absorbent system which it scarcely deserves; and in acknowledging that its ill effects are not well marked when used in an appropriate manner, we also indicate that its curative influence is feeble or uncertain. Our tonics rarely produce great injury. Iron and the vegetable bitters enter largely into the composition of fancy medication, and can be given indiscriminately for anv length of time without any great harm, generally without any very great good. Iron has its positive advantages, and is valuable. Bark is extensive in its applicability, and is certainly controlling in some forms of disease. The active principle of bark may act as a distnrbing element; and iron even may not be wholly harmless.

Cathartics are valuable, and aid one of the most important natural secretions; but how largely may they all be regarded as disturbing agents, often working much more harm than good We might pass over the whole list of the *materia medica*. and all important valuable remedies would be seen to possess some disturbing elements. This does not show them less useful or important in the treatment of disease; it is nothing against a drug that it is capable of evil, is a violent poison in sufficient quantity. Indeed, all our valuable remedies may possess poisonous or injurious qualities; and to the point that medicines are generally disturbing agents, we would direct attention. Nature rebels against, and attempts the removal of, disease, or at once institutes efforts for overcoming its effects. The attentive observer of nature in disease will learn much in therapeutics.

The disturbing influence of medicine has contributed largely to the prevalence of the various forms of imposition now practiced upon mankind under the guise of medical reform, seeking some plan of caring for the sick, which did not make them still sicker. A system of medication which, if it could do no good, should at least do no harm, has been welcomed by many, and regarded as an improvement and a reform. Physicianshave, heretofore, and, I am sorry to say, do still torment sick people with quite unnecessary and often useless measures of relief. They order, with thoughtless indifference, emetics, active cathartics, and, above all, and worse than all, blisters and counter irritants. The moral force of the physician, and the charm of thinking that something is being done for them, may, for a time, suffice to keep up the delusion, and make them believe that the "heroic" measures were indispensable to recovery; but many a family have been driven from their old physician to some inert practitioner innocent of any rational views of disease, by blisters, emetics, violent cathartics, forced swabbings of the throat, and other painful and useless methods of treating disease. The inquiry is at once made, are not these means necessary to successfully combat and arrest the progress of acute maladies themselves more painful than the measures of relief? To this question we answer, no, no; a thousand times, no. Whoever advises these disturbing measures of treatment, not only injures his own reputation and business, but hastens the time when his friends will join the army of revolt, and seek other and less painful modes of treating their diseases.

Pharmacy has been so greatly improved that regular medicine is palatable if the physician notices the best mode of prescribing it. And since sugar medication and water medication is now almost wholly abandoned as useless, and has given place to disguised medicine, we should hope that, finally, *rational medication* may take the place which rightfully belongs to it, both in the practice of physicians and in the confidence and respect of an intelligent public.

These hasty remarks are made with the sole view of directing attention to points of great importance to the profession. First, the idea of depending upon nature, which, under favorable circumstances, is wholly adequate to restoration, in almost all cases capable of such termination: and second, the necessity of avoiding the harsh and aggressive measures of treatment wherever this is consistent with the satisfactory progress and termination of disease. The Homœopathic delusion is the offspring—the reaction of violent and aggressive medication; and wherever it has been accepted by the public, it has been mainly because more agreeable, though in its original purity it was most obviously inert; and now, as an unmeaning name, is a convenient cover for all sorts of medication.

